# Title: efficacy of a food parenting intervention for mothers with low income to reduce preschooler’s solid fat and added sugar intakes: a randomized controlled trial

**DOI:** 10.1186/s12966-018-0764-3

**Published:** 2019-01-17

**Authors:** Jennifer O. Fisher, Elena L. Serrano, Gary D. Foster, Chantelle N. Hart, Adam Davey, Yasmeen P. Bruton, Linda Kilby, Lisa Harnack, Karen J. Ruth, Alexandria Kachurak, Hannah G. Lawman, Anna Martin, Heather M. Polonsky

**Affiliations:** 1 Center for Obesity Research and Education, College of Public Health, Temple University, 3223 N. Broad Street, Suite 175, Philadelphia, PA 19140 USA; 2 Department of Human Nutrition, Foods, and Exercise, Virginia Tech, 327 Wallace Hall, Blacksburg, VA 24061 USA; 3 Weight Watchers International, 675 6th Ave, New York, NY USA; 4 Weight and Eating Disorders Program, University of Pennyslvania, Pennyslvania, USA; 5 Department of Behavioral Health and Nutritio, University of Deleware, 385 McDowell Hall, Neward, Newark, DE 19716 USA; 6 Department of Obstetrics & Gynecology, Division of Urogynecology, Duke University at Patterson Place, 5324 McFarland Drive, Suite 310, Durham, NC 27707 USA; 7 LDN. NORTH Inc, Philadelphia WIC program, 1300 W Lehigh Avenue, Philadelphia, PA 19132 USA; 8 Division of Epidemiology and Community of Public Health, School of Public Health, University of Minnesota, 1300 S 2nd Street, Room 300 West Bank Office Building, Minneapolis, MN 55454 USA; 9 Biostatistics Facility, Fox Chase Cancer Center, 333 Cottman Avenue, Philadelphia, PA 19111 USA; 10 Division of Chronic Disease Prevention, Philadelphia Department of Public Health, 1101 Market Street, 9th Floor, Philadelphia, PA 19107 USA; 11 Providence Health and Services, Center for Outcomes Research & Education, 5251 NE Gilsan Street, Bldg A, Portland, OR 97213 USA

**Keywords:** Low-income, Food parenting, Authoritative, Dietary intervention, Solid fats, Added sugars, Preschooler, Prevention, Randomized controlled trial

## Abstract

**Background:**

Few interventions have shown efficacy to influence key energy balance behaviors during the preschool years.

**Objective:**

A randomized controlled trial (RCT) was used to evaluate the efficacy of Food, Fun, and Families (FFF), a 12 week authoritative food parenting intervention for mothers with low-income levels, to reduce preschool-aged children’s intake of calories from solid fat and added sugar (SoFAS).

**Methods:**

Mothers were randomly assigned to receive FFF (*n* = 59) or to a delayed treatment control (*n* = 60). The primary outcome was children’s daily energy intake from SoFAS at the end of the 12 week intervention, controlling for baseline levels, assessed by 24-h dietary recalls. Secondary outcomes included children’s daily energy intake, children’s BMI z-scores, and meal observations of maternal food parenting practices targeted in FFF (e.g. providing guided choices).

**Results:**

Participating mothers were predominantly African American (91%), with 39% educated beyond high school and 66% unemployed. Baseline demographics and child SoFAS intakes did not differ by group. Lost to follow-up was 13% and did not differ between groups. At post-intervention, FFF children consumed ~ 94 kcal or 23% less daily energy from SoFAS than children in the control group, adjusting for baseline levels (307.8 (95%CI = 274.1, 341.5) kcal vs. 401.9 (95%CI = 369.8, 433.9) kcal, FFF vs. control; *p* < 0.001). FFF mothers also displayed a greater number of authoritative parenting practices when observed post-intervention with their child at a buffet-style meal (Wilcoxon z = − 2.54, *p* = 0.012). Neither child total daily energy intake nor BMI z-scores differed between groups post-intervention.

**Conclusions:**

Findings demonstrate the initial efficacy of an authoritative food parenting intervention for families with low-income to reduce SoFAS intake in early childhood. Additional research is needed to evaluate longer-term effects on diet and growth.

**Trial registration:**

Retrospectively registered at ClinicalTrials.gov: #NCT03646201.

## Background

Surprisingly few interventions to date have demonstrated efficacy for influencing key energy balance behaviors during early childhood [1], a critical period for the development of healthy habits [2, 3]. The need for effective early intervention is particularly pressing for young children from families with low-income that may be disproporiately affected by poorer diet quality [4, 5] as well as obesity [6]. Solid fats, which are high in saturated and trans fats, as well as added sugars (SoFAS) are key dietary targets because they contribute “excessive” or “empty” (i.e. energy with few to no other nutrients) calories [7, 8] to children’s diets, have been linked to obesity and cardiovascular disease risk [9, 10], and are currently consumed by children at levels that well-exceed recommendations [11, 12]. US preschoolers, for instance, consume roughly 13 teaspoons (53 g) of added sugar each day, which is more than double recent recommendations (6 teaspoons per day or 24 g) from the American Heart Association [10]. The emphasis of current US Dietary Guidelines on healthy eating patterns that limit calories from saturated fat, added sugars, and sodium [8], along with recent calls to reduce added sugar intakes among children [10] highlight the need for effective public health efforts to reduce children’s consumption of “empty” calories.

Parents neccesarily play a central role in addressing children’s SoFAS intake given their influence on types and amounts of foods made available to children, their roles as social models of eating behavior, and their parenting approach to socializing children’s eating behaviors [13]. The need to meaningfully engage parents in prevention efforts is reflected in the recent proliferation of family-based interventions; a recent review identified 119 obesity prevention interventions targeting children 2–17 years, with 43% focused on preschool aged children [14]. Of those involving preschoolers, a number have targeted one or more specific types of high SoFAS food (e.g. sugar sweetened beverages(SSB)) [15–18], often in conjunction with other energy balance behaviors (e.g. screen time). However, none have focused more globally on young children’s “empty” calorie consumption globally, across the entire diet.

While family-based prevention implicitly targets parents as agents of change, few dietary interventions have used a theoretical lens that emphasizes parenting and its influence on child development. Of 119 family-based obesity prevention interventions recently reviewed, only 17% drew from parenting theory [14]. The lack of theory-driven research on parenting has been identified as a barrier to implementing effective interventions to improve children’s dietary intake [19]. Current theoretical perspectives hold that authoritative approaches to feeding children will be most likely to foster optimal eating (and weight) outcomes [20, 21]. Authoritative parenting practices provide structure such as limit setting and making healthful foods available and accessible to children, as well as autonomy supportive practices, which include praise, responsiveness to child appetite cues, and role modeling [21]. Despite the current emphasis on authoritative food parenting, evidence of beneficial effects on children’s dietary energy balance behaviors from controlled trials is surprisingly limited, particularly in preschool-aged children.

The aim of this research was to evaluate the efficacy of the 12-week *Food, Fun, and Families* (FFF) parenting intervention for reducing children’s consumption of “empty” calories from SoFAS using an RCT design. FFF taught mothers authoritative parenting skills for reducing children’s daily energy intake from SoFAS, which was the primary outcome at post-intervention evaluated relative to the control group, adjusting for baseline values. Secondary aims were to increase use of authoritative food parenting practices targeted in FFF (e.g. limiting high SoFAS foods, using child size dishware), and to lower children’s daily energy intakes from added sugars (kcal), daily energy intakes from solid fats, total daily energy intakes, and BMI-for-age z-scores at post-intervention relative to the control group.

## Methods

### Overview of design

FFF was evaluated using a group-based 12 week RCT conducted in a university clinic-based setting between January 2013–September 2015. Mothers were randomly assigned to receive the FFF intervention or to a delayed treatment control group by the research coordinator using a randomized permuted blocks procedure where 16–24 mothers were divided into two groups of 8–12 across six successive waves of enrollment to ensure 1:1 treatment allocation. Sequentially numbered envelopes were created by the methodologist (AD) to create and conceal the allocation sequence for each block. Mothers in the FFF group attended weekly group intervention sessions for twelve weeks, whereas those in the control group were offered FFF after completion of the 12 week post-intervention measures. Primary and secondary outcomes were assessed at baseline and at the end of the 12 week intervention, with the exception of meal observations which were made only at post-intervention.

Mothers were required to complete at least 2 of the 3 dietary recalls at baseline to be randomized and at post-intervention to be included in outcome analyses. The study team was blind to treatment allocation prior to randomization. Dietary assessment staff who collected the primary outcome data and the research assistant who coded meal observations of authoritative food parenting practices (a secondary outcome) were blind to group assignment following randomization. A sample size of *n* = 50 mothers per group was targeted to provide power > 0.80 to detect a difference of 150 SoFAS calories between groups post-intervention; this difference is clinically meaningful for obesity prevention [22] and reflects an approximately 30% reduction in current estimates of daily discretionary calorie intakes among preschool aged children [11].

### Participants

Participants were the biological mothers of 3 to 5 year-old children, who reported primary responsibility for feeding the child at home, and received or were income-qualified to receive the Supplemental Nutrition Assistance Program (SNAP). Participants were excluded if the target child had severe food allergies, special dietary restrictions, chronic medical conditions (e.g. diabetes), or had a developmental disorder (e.g. autism) that influenced eating and/or weight.

Recruitment was primarily in-person at Women, Infants, and Children (WIC) offices in Philadelphia, PA. A project staff member provided a brief description of the study and collected contact information from potentially interested participants in the office waiting area. Study flyers/ads were also posted in community settings and online forums (e.g. Craigslist). Interested parties were screened for eligibility by telephone using a written script. Mothers provided written consent for their own and their child’s participation. Each family in both groups was compensated a total of $400 for attendance at weekly sessions (FFF group only), participation in assessments, and travel to/from the research center. The study was approved by and conducted in accordance with the ethical standards of the Institutional Review Board at Temple University. FFF was developed as a nutrition education program for delivery in the US Department of Agriculture (USDA) Supplemental Nutrition and Food Assistance Program. Because there was no precedent for registering USDA-funded nutrition education programming with ClinicalTrials.gov at the time the work was conducted (2013–2015), we registered the trial retrospectively (www.clinicaltrials.gov: #NCT03646201). The primary outcome of children’s daily energy from SoFAS reflects the USDA’s interest in nutrition outcomes and is presented in the manuscript as originally proposed.

### Intervention – *Food, Fun, and Families Program*

#### Formative research

Twelve weekly FFF sessions were developed, manualized, and pilot tested for acceptability/feasibility using a single arm trial with 9 women from low-income backgrounds. Formative research was used to identify key “frames” for aligning FFF dietary goals with mother’s aspirations around parenting and feeding children in families with low income levels [23–25]: 1) connecting with children and building lasting family bonds at mealtimes; 2) preventing (perceived) hyperactivity and tooth decay by limiting children’s sugar intake; 3) using feeding to teach children life lessons about limit setting and structure; and 4) being responsive to children during mealtimes to guide decisions about portions.

#### Intervention framework

FFF content was guided heavily by current theoretical perspectives on authoritative food parenting [20, 21] that emphasize the use of structure and autonomy support in feeding. Feeding pratices that promote structure were emphasized included establishing eating routines, setting limits, and providing children with guided choices (i.e. allowing the child to choose between two or three options that the parent determines are acceptable) [21, 26], whereas a number of practices promoting autonomy support were also emphasized including effective praise, responsiveness to child hunger and satiety cues, and parental modeling [21, 26]. FFF modules also targeted aspects of the family eating environment known to influence children’s intake of energy-dense foods, including the use of child size dishware/glasses [27, 28], offering child size portions [29], using the 1 tablespoon per year of life rule of thumb [30], and stimulus control (i.e., reducing the availability of high SoFAS foods) [26]. Food parenting skills were used to help mothers place limits on “WHOA” foods and beverages (e.g. SSB, desserts, candy, potato chips) and alternatively encourage offering healthier “GO” choices (e.g. water, low-fat milk, fruits, vegetables, pretzels), consistent with the focus of the current dietary guidance to shift to dietary patterns to include healthier alternatives [8]. Particular attention was given to SSB [31] and the quality of foods offered as snacks [23, 32, 33] which are significant sources of SoFAS in young children’s diets.

#### Intervention session components

Each 60-min weekly in-person group session used core behavioral change techniques to facilitate adherence including goal setting and planning (i.e. group problem-solving around challenges to achieving weekly goals), feedback and monitoring (i.e.self-monitoring of FFF weekly goals and target behaviors), antecedents (i.e. restructuring the physical environment), comparison of behavior (i.e. group discussion of success and challenges), natural consequences (i.e. emotional consequences of parenting), and social reward (i.e.reinforcement/praise of progress by interventionists) [34]. Sessions began with a group discussion of progress on achieving the previous weeks’ goals and collective problem-solving around challenges (SHARE; 25 min), followed by presentation of new content and interactive demonstrations (GROW; 25 min), and finally a discussion of the next week’s goals (GO; 5 min). Each weekly module contained a resource kit for the interventionist including facilitator guide, demonstration instructions, and participant handouts. Sessions were led in a university clinic-based setting by one of two graduate-level interventionists who received training from clinical psychologists with expertise in behavioral interventions (GDF, CH).

### Outcome measures

***Child daily energy from SoFAS*** was assessed using three 24-h dietary recalls administered over ~ 2 week periods at baseline and post-intervention. Two weekday and one weekend recalls were collected via telephone by trained and certified staff at the Nutrition Coordinating Center, University of Minnesota, Minneapolis, MN using Nutrition Data System for Research (NDSR) software (versions 2012, 2013, and 2014) [35]. NDSR software uses multiple-pass interview methods to collect dietary recalls in 5 distinct passes (initial list all foods and beverages, review for completeness, amount consumed and preparation, forgotten food probes, final review). Food amount booklets were provided to assist participants in reporting portion sizes. Meal type, location, and who fed the child were noted for each food and drink reported. A comprehensive set of quality assurance procedures were followed, which included procedures implemented during recall collection (e.g. asking participants to confirm unusually small and large food portions) as well as after collection (e.g. reviewing completed recalls to identify data entry errors such as entering beverages in ounces rather than fluid ounces. Children’s daily energy intake of SoFAS, total energy, added sugar, and solid fats were estimated reported food intake to the Nutrition Coordinating Center database containing nutrient information for 18,000 foods and 8000 brand name products [36, 37].

***Child and maternal weight status*** were assessed using measured height and weights in the laboratory obtained using procedures described by Lohman, Roche, and Martorell [38]. Height and weight were measured in duplicate to the nearest 0.1 cm with a wall-mounted stadiometer (Holtain Limited, Harpenden, Pembs, UK) and to the nearest 0.1 kg using an electronic balance (Detecto, Model 758C, Webb City, MO), respectively. Measurements within 0.2 units were averaged; in cases where values differed by > 0.2 units, a third measurement was used to replace the most discrepant value. Child BMI-for age z-scores were calculated using Centers for Disease Control and Prevention reference data [39]. Additionally, child BMI (kg/m^2^), child BMI percentiles were calculated for descriptive purposes, with child overweight/obesity defined as BMI-for-age ≥ 85th percentile [40]. Maternal BMI (kg/m^2^) was calculated and categorized for descriptive purposes using standard criteria [41]: overweight (BMI 25–29.9) and obesity (BMI ≥30).

***Meal observations of authorative food parenting practices*** were made in a laboratory setting to assess authoritative food parenting targeted in FFF at post-intervention. A digital video recording was made of mother-child dyads at a buffet-style meal in a private room that included a buffet table and a separate table at which to eat. The buffet included an assortment of FFF targeted “GO” foods (i.e. green beans, beets, sliced peaches, grapes) and beverages (i.e.1% milk, water) as well as “WHOA” foods (i.e. chocolate chip cookies, apple pie) and drinks (i.e. orange soda, fruit punch) based on fat/sugar content as well as other foods not targeted explicitly in the intervention (i.e. chicken nuggets, mashed potatoes). Roughly 4 times the manufacturer’s serving size of each food/beverage was provided in serving containers. A tablespoon was placed in each serving bowl. Both child and adult size dishware were provided without instruction: child (7.5 in. diameter plates, 6 oz. cups) and adult sizes (10 in diameter plates and 12 oz. cups).

Mothers were instructed to avoid giving the child anything to eat or drink for 2 h prior to the observation. At the beginning of the 25 min observation, the mother was instructed to serve/eat as much or as little as they desired for themself and their child, and were instructed to eat as they normally would during meal times. After the completion of the RCT data collection, raters used digital recordings to code the presence or absence of 9 authoritative food parenting practices targeted in FFF: 1) sits with the child during meal without distractions (e.g. cell phones); 2) use of child size plates and glasses (vs. adult size); 3) serves only “GO” beverages of milk or water (vs. sugar sweetened beverages); 4) offers child size portions (i.e. follows 1 tablespoon per year of life [30], as determined by the number of tablespoons served to child, based on age); 5) limits “WHOA” foods offered (i.e. no more than 1 small cookie or piece of pie); and 6–9) use of 4 responsive feeding strategies (i.e. encourages trying, provides guided choices (e.g. specifies options for child), asks child about hunger/fullness, and uses praise). Total scores ranged from 0 to 9. A single rater coded all observations; inter-rater agreement on the total score was good for a randomly selected 20% of observations coded by a second rater (ICC = 0.90, *p* < 0.01).

### Other measures

Demographic data were self-reported and included maternal and child race/ethnicity, maternal education, marital status, and employment, and family participation in U.S. federal nutrition and food assistance programs.

### Analyses

Stata Version 15.1 (College Station, TX) was used for all statistical analyses. Statistical significance was inferred by *p*-values < 0.05. Descriptive statistics were generated for demographic variables; t-tests (for continuous variables) and Chi-square tests or Fisher’s exact tests (for categorical variables) were used to evaluate baseline differences across groups.

Analysis of covariance (ANCOVA) was used to evaluate the post-intervention differences between groups in the primary outcome of total daily energy from SoFAS (kcal/d), with an as-treated design; cases missing daily SoFAS energy at post-intervention were excluded from the outcome analyses. Models were adjusted for baseline levels to provide more exact estimates of treatment effects, by allowing each individual to effectively serve as their own control. Between group differences in children’s total daily energy from SoFAS were also evaluated based on only those occasions where the mother reported feeding the child. ANCOVA, controlling for baseline values, was also used to evaluate post-intervention differences in secondary outcomes involving child dietary intake (e.g. daily energy intake, daily energy from solid fats, daily energy from added sugars) and child weight (e.g. BMI z-scores). Backwards elimination (*p* < 0.10) using linear regression was used to evaluate child gender, age, race, maternal age, education, marital status, and participation in federal nutrition and food assistance programs as potential covariates in predicting the primary outcome of daily energy from solid fat and added sugars; of these only maternal age was retained and included in all models. Child BMI and BMI percentiles are presented for the purposes of comparison with BMI z-score results. Effect sizes for dietary and weight outcomes were calculated using adjusted means using Cohen’s *d* [42].

Wilcoxon rank-sum test was used to compare the distribution of total number of authoritative food parenting practices observed a buffet style meal at post-intervention across groups. Chi-Square tests were used to test the proportion of mothers in each group displaying each of 9 authoritative food parenting practices.

## Results

**Fig. 1:**
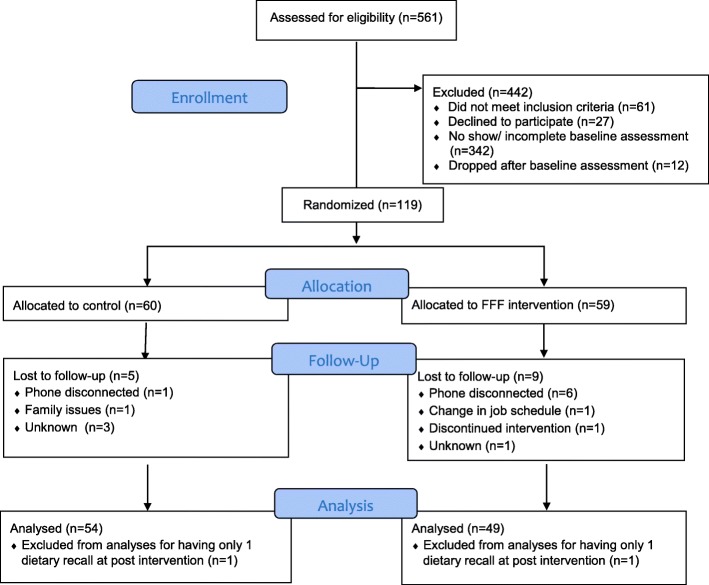
Consort diagram

*Randomization, attendance, and differential non-response*. As depicted in the Figs. 1, 119 mothers were randomized to either control (*n* = 60) or FFF intervention groups (*n* = 59). As shown in Table 1, demographic characteristics did not differ between control and FFF groups. All participating caregivers were mothers, most of whom (~ 91%) reported being Black/African American. The majority of mothers reported being single (78.2%) and unemployed (65.8%). Education was varied with close to 40% reporting education beyond high school. Most mothers reported participation in WIC (90.8%) and SNAP (87.4%) federal food and nutrition assistance programs. Among participating children, gender was roughly split with slightly more females (54.6%) than males (45.4%). More than 50% of mothers had obesity and almost 30% of children had either overweight or obesity at baseline.

**Table 1:** Baseline demographics^a^

	Total (*n* = 119)		Control (*n* = 60)		FFF intervention (*n* = 59)		*p*-value
	*n*	%	*n*	%	*n*	%	
Mother							
Age (y; mean (SD))	29.8 (7.1)		30.0 (6.9)		29.5 (7.3)		0.71
Race/ethnicity							
Black/African American	108	90.8	57	95.0	51	86.4	0.11
Other	11	9.2	3	5.0	8	13.6	
Marital status							
Single	93	78.2	49	81.7	44	74.6	0.66
Married/living together	22	18.4	9	15.0	13	22.0	
Other	4	3.4	2	3.3	2	3.4	
Education							
< HS	31	26.0	17	28.3	14	23.7	0.49
HS graduate/GED	42	35.3	23	38.3	19	32.2	
> HS	46	38.7	20	33.3	26	44.1	
Employment status							
Unemployed	77	65.8	39	66.1	38	65.5	0.95
Employed for wages	40	34.2	20	33.9	20	34.5	
Weight status^b^							
Normal	26	22.4	13	22.4	13	22.4	0.67
Overweight	30	25.9	13	22.4	17	29.3	
Obese	60	51.7	32	55.2	28	48.3	
Program participation (# participating)^c^							
WIC	108	90.8	56	93.3	52	88.1	0.33
SNAP/EBT	104	87.4	53	88.3	51	86.4	0.76
Head Start	46	38.7	26	43.3	20	33.9	0.29
Child							
Age (y; mean (SD))	3.7 (0.8)		3.8 (0.1)		3.6 (0.1)		0.09
Gender							
Female	65	54.6	29	48.3	36	61.0	0.17
Male	54	45.4	31	51.7	23	39.0	
Weight status^d^							
Normal	81	69.8	38	65.5	43	74.1	0.57
Overweight	22	19.0	13	22.4	9	15.5	
Obese	13	11.2	7	12.1	6	10.4	

^a^Between group differences in demographic characteristics were evaluated using chi-square and Fisher’s exact tests for categorical variables and t-tests for continuous variables. BMI was not recorded for 3 mothers/children and 2 mothers did not report employment status at baseline

^b^Overweight = BMI (kg/m^2^) 25.0 to < 30.0; Obese = BMI (kg/m^2^) ≥ 30.0

^c^WIC = Women’s Infant and Children Program, SNAP/EBT = Supplemental Nutrition Assistance Program/Electronic Benefits Transfer

^d^Overweight = BMI-for-age 85th to <95th percentiles; Obese = BMI-for-age ≥ 95th percentile

Among the 59 mothers participating in FFF, mean attendance was 6.4 (SD = 4.2) out of 12 weekly sessions, with 18 (30.5%) mothers attending fewer than 25% of sessions, 4 (6.8%) attending 25–49% of sessions, 14 (23.7%) attending 50–74% and 23 (40%) attending 75% or more of the sessions during the 12 week intervention.

As shown in Figs. 1, 14 (12%; 9 FFF intervention, 5 control) of the 119 mothers who were randomized were lost to follow up; additionally 1 FFF intervention and 1 control mother were excluded from primary outcome analyses for failing to meet the criterion of providing at least 2 of the 3, 24 h dietary recalls at post-intervention. Attrition (10 FFF vs. 6 control) did not differ by group (*p* = 0.27, Chi-square test). Of the 103 cases (54 control and 49 FFF) with primary outcome data at both timepoints, an additional 9 (5 control, 4 FFF) were missing data on child weight status and 16 (6 control, 10 FFF) were missing meal observations made at a separate visit at the end of the intervention.

*Dietary intake*. At baseline, 98% of FFF mothers and 95% of control mothers provided 3 dietary recalls; the remainder provided 2 recalls. Similarly, at post intervention, 96% of the 49 FFF mothers who completed post-intervention assessments and 98% of the 54 control motheres provided 3 dietary recalls, with the remainder of completers providing 2 recalls.

At baseline, mothers reported having responsibility for child feeding (i.e. providing food) at 78.2% of children’s daily eating occasions and there were no differences between groups. Children were reported to consume, on average, 375 kcal/d (approximately 29% of total daily energy intake) from SoFAS and there was no differrence between groups (Table 2). At post-intervention, FFF children consumed ~ 94 kcal or 23% less daily energy from SoFAS than children in the control group, adjusting for baseline levels (307.8 (95%CI = 274.1, 341.5) kcal vs. 401.9 (95%CI = 369.8, 433.9) kcal, FFF vs. control; *p* < 0.001). Most (79.2 kcal) of the 94 kcal difference between groups was consumed at eating occasions where the mother reported responsibility for feeding the child. Between group differences at post intervention were seen separately for children’s daily energy from added sugars and from solid fats; FFF children consumed ~26% less energy from added sugars and 22% less energy from solid fat than controls at post-intervention; both *p* < 0.01). Total daily energy intake among FFF children was estimated to be 91 kcal/d lower than controls at post-intervention; however, this difference was not statistically significant.

**Table 2:** Child dietary intakes at baseline and post-intervention by group, with adjustment for baseline levels

		Control		FFF Intervention				
	*n*	Mean (SD)	Adj Mean^e^ (95%CI)	n	Mean (SD)	Adj Mean (95%CI)	*p*-value	Adj Cohen’s d^f^
*Primary Outcome*								
SoFAS (kcal)								
Baseline	60	392.9 (159.0)		59	356.4 (139.3)			
Post-intervention^g^	54	408.8 (150.4)	401.9 (369.8, 433.9)	49	300.2 (100.4)	307.8 (274.1, 341.5)	< 0.001	−0.63
*Secondary Outcomes* SoFAS (kcal), occasions fed by mother								
Baseline	60	317.1 (164.7)		59	288.9 (145.4)			
Post-intervention	54	331.6 (156.8)	324.6 (290.3, 358.9)	49	237.6 (121.6)	245.4 (209.3, 281.4)	< 0.001	−0.51
SoFAS (% of daily energy)								
Baseline	60	28.6 (7.0)		59	28.7 (5.9)			
Post-intervention	54	30.0 (7.3)	29.9 (28.2, 31.6)	49	24.7 (5.5)	24.7 (22.9, 26.5)	< 0.001	−0.79
Added sugar (kcal)								
Baseline	60	180.7 (87.3)		59	177.7 (95.3)			
Post-intervention	54	195.1 (97.7)	193.7 (171.5, 215.9)	49	141.9 (77.6)	143.5 (120.2, 166.8)	0.003	−0.55
Solid fat (kcal)								
Baseline	60	212.2 (99.0)		59	178.7 (69.5)			
Post-intervention	54	213.7 (93.2)	209.1 (188.5229.7)	49	158.2 (59.4)	163.3 (141.6184.9)	0.003	−0.53
Total daily energy (kcal)								
Baseline	60	1344.2 (339.7)		59	1223.3 (321.7)			
Post-intervention	54	1367.5 (382.6)	1334.8 (1254.6, 1415.0)	49	1207.6 (285.9)	1243.6 (1159.3, 1327.9)	0.13	−0.10

^e^Adjusted for baseline value, using ANCOVA

^f^Calculated using adjusted means

^g^Missingness (*n* = 16) on the primary outcome did not differ by group (*p* = 0.27, Chi-square test)

**Fig. 2:**
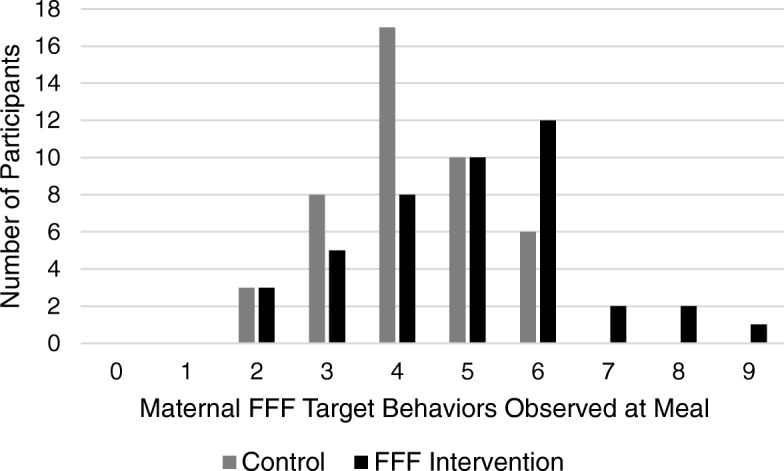
Meal observation of authoritative food parenting practices at post-intervention by group. Total number of authoritative food parenting pratices observed when mother-child dyads were seen at a buffet-style meal, post-intervention (possible scores 0–9). A Wilcoxon rank-sum test (Z = −2.54, *p* = 0.012) revealed that the distribution of authoritative food parenting pratices at post-intervention among mothers in the FFF intervention group (*n* = 43) was shifted to the right, relative to mothers in the Control group (*n* = 44)

*Meal observations of authoritative food parenting practices.* As shown in Fig. 2, the distribution of authoritative food parenting practices observed post-intervention at a buffet style meal differed between groups (Wilcoxon rank-sum, Z = − 2.54, *p* = 0.012), with scores among FFF mothers showing a shift towards greater use of authoritative pratices. A greater proportion of FFF mothers than controls were observed to use child vs. adult size dishware (84% vs. 60%, FFF vs. control; *p* < 0.05) and to offer children only GO beverages (i.e.low-fat milk or water) at the meal (49% vs. 23%, FFF vs. control; *p* < 0.05) compared to mothers in the control group. The other individual pratices did not differ between groups (data not shown).

*Child weight status*. As shown in Table 3, neither child BMI-for-age z-score nor child BMI percentile differed between groups at post-intervention, when adjusting for baseline values.

**Table 3:** Child weight status at baseline and post-intervention by group, with adjustment for baseline levels

	Control			FFF Intervention				
	N^h^	Mean (SD)	Adj Mean^i^ (95%CI)	N	Mean (SD)	Adj Mean (95%CI)	*p*-value	Cohen’s d^j^
BMI (kg/m^2^)								
Baseline	58	16.5		58	16.5		0.72	0.02
		(1.8)			(2.4)			
Post-intervention	49	16.5 (1.6)	16.4 (16.2, 16.6)	45	16.2 (1.3)	16.4 (16.2, 16.6)		
BMI-for-age z-score								
Baseline	58	0.60 (0.98)		58	0.48 (1.20)			
Post-intervention	49	0.64 (0.90)	0.57 (0.45, 0.68)	45	0.49 (0.83)	0.60 (0.48, 0.73)	0.66	0.04
BMI-for-age percentile								
Baseline	58	66.2 (26.3)		58	61.9 (28.7)			
Post-intervention	49	67.9 (24.7)	66.2 (62.7, 69.8)	45	64.3 (25.1)	67.3 (63.6, 70.9)	0.69	0.04

^h^Reflects exclusion of 1 biologically implausible value (BMI z-score = 5.79 [51]) at baseline and missing values

^i^Adjusted for baseline value, using ANCOVA

^j^Calculated using adjusted means

## Discussion

This research is the first early childhood randomized controlled trial to examine efficacy of intervening on food parenting strategies to broadly reduce preschool aged children’s exposure to and intake of “empty” calories from SoFAS [19] across the entire diet. Results showed that the FFF intervention produced significant decreases in children’s daily consumption of SoFAS and resulted in positive shifts in authoritrative parenting pratices relative to the controls. These results are noteworthy given that FFF focused on dyads who are typically underrepresented in family-based prevention research [14] and yet may be at risk for pooer diet quality [4, 5]. In general, the findings suggest that interventions targeting authoritative food parenting can be efficacious in reducing consumption of “empty” calories in early childhood.

To date, the majority of nutrition interventions specifically targeting child diet using parents as primary change agents have tended to involve older children (6–11 y) and used indirect methods of engagement, such as newsletters, health fairs, and home-based assignments [19]. The FFF intervention adds to this literature by drawing heavily on current food parenting theory to directly engage mothers to reduce children’s exposure to and intake of “empty” calorie foods. A number of family-based obesity prevention trials conducted in the past decade have directly engaged parents around food parenting and/or the family food environment [14]. However, most early childhood prevention trials have focused on select aspects of diet quality, most often fruit and vegetable intake [17, 43–47], with fewer intervening on and/or demonstrating effects on selected types of high SoFAS foods, like children’s intakes of processed snack foods [47] and sugar sweetened beverages [15–18, 46]. The present study is novel in that is among the first controlled trials to successfully intervene on food parenting to reduce children’s intake of “empty” calories across the entire diet, in a sample of mother-child dyads at increased risk of poor diet quality and obesity. Reductions in SoFAS intake among FFF children primarily came from eating occasions at which the mother reported providing the food. FFF mothers also showed greater use of authoritative feeding practices at post-intervention relative to controls. Collectively, these findings suggest that FFF was effective in teaching mothers how to identify high SoFAS foods and use authoritative practices to reduce children’s exposure and consumption. That the use of child size plates and offering “Go” beverages (i.e. milk, water) were the only two practices that individually differed between groups indicates that these practices may be particularly amenable to change and also indicates heterogeneity in the adoption of other authoritative practices by FFF mothers. Future research should seek to identify which authoritative strategies are most critical for influencing SoFAS intake and most likely to be adopted by caregivers.

Intervention effects on children’s daily SoFAS intake at post-intervention were attributable to decreases in children’s intake of both added sugar and solid fats. From a translation perspective, however, public health efforts to reduce added sugars may be easier than solid fats in that they come from a relatively narrow range of foods in children’s diets. For instance, among US children 2–18 y, the top 5 food sources of solid fat represent 44.1% of intake, whereas the top 5 sources of added sugar represent 72% of intake, with the largest contribution from sugar sweetened beverages (~ 47%) [48]. Further, among preschoolers, close to 40% of daily added sugar intakes come from snacks [33]. Additional research is needed to understand the effectiveness of specifically targeting added sugars from snacks for reducing children’s overall intake of “empty” calories.

While FFF proved to be effective for improving an important dietary outcome for prevention in a high risk population, implications for children’s total energy intake and weight gain are less clear. There are a number of plausible interpretations of the null effects of FFF on total energy intake, child BMI z-score and other weight outcomes. First, it is possible that children may have compensated for reductions in high SoFAS foods by increasing intakes of others. In this case, targeting high SoFAS foods alone may not be sufficient to alter patterns of children’s total energy intake. It is important to note, however, that the 91 kcal difference in daily energy intake between groups at post-intervention, while not statistically significant, is clinically significant within the context of obesity prevention [22] and of a similar magnitude to the significant observed difference in children’s SoFAS intake between groups (94 kcal). In terms of the null effect on weight status, it is possible that mothers’ reports did not accurately reflect children’s actual dietary intake; self-report biases in dietary assessment are well established. Alternatively, it may be that changes in maternal behavior and resulting reductions in child SoFAS intake were not immediate, such that longer periods are required to detect preventive effects on excessive weight gain. Additional research is needed to evaluate these and other substantive explanations.

Several limitations of this research merit consideration. The primary outcome, while measured using 2–3 dietary recalls at each time point collected using rigorous multiple-pass methods, is based on caregiver report and may have been subject to reporting bias [49]. That the meal observations of maternal FFF target behaviors are consistent with the dietary results lends support to the integrity of the main findings but could also reflect demand bias. At a minimum, the results suggest that the FFF intervention was successful in helping mothers to identify high SoFAS foods in their children’s diets and relevant parenting behaviors for reducing children’s exposure. It is important to note that the observational data were only collected post-intervention and therefore cannot rule out the possibility of baseline differences between groups or speak to change over time. Finally, while findings demonstrate efficacy of FFF for reducing reported SOFAS among families with low-income who have been notably difficult to reach in prevention efforts to date, the percent of individuals who agreed to participate was modest (24%). Further, while retention (87%) was very similar to rates reported in obesity prevention trials with children from low-income families [50], attendance was variable and quite low for close to a third of mothers. These observations clearly underscore the need for prevention efforts to develop strategies for better reaching and engaging vulnerable populations, including families with low-income [50].

## Conclusions

In conclusion, the findings of this research demonstrate initial efficacy of the FFF parenting intervention for reducing young children’s intake of “empty” calories from SoFAS among families with low levels of income. Results demonstrate the promise of authoritative food parenting interventions for addressing key energy balance dietary behaviors in vulnerable populations. Specifically, these findings suggest that addressing parenting strategies and skills are important for achieving nutritional targets that have traditionally been the focus of nutrition education interventions [19]. The use of formative work to align intervention goals with maternal aspirations for parenting and feeding children may be important for engaging mothers in prevention efforts, particularly in populations that have been historically understudied and hard to reach [23–25]. This work suggests benefits of aligning nutritional and feeding recommendations with broader maternal aspirations around child development and the parent-child relationship. Additional research is needed to evaluate the effectiveness and adoption of FFF within community settings, including nutrition assistance and education programs reaching parents of young children with low-income, such as the Supplemental Nutrition Assistance Education Program, WIC, and Head Start. Longer term research is also needed to understand whether increases in intervention dose, the use of booster sessions, and/or additional intervention strategies (e.g. home food environment, parenting) could increase extent to which reductions in SoFAS intake translate to reductions in daily energy intake and the prevention of obesity among children.

## Abbreviations

FFF: Food, fun, and families.
SoFAS: Solid fat and added sugar.
BMI: Body mass index.
SSB: Sugar sweetened beverages.
RCT: Randomized controlled trial.
SNAP: Supplemental Nutrition and Food Assistance Program.
USDA: United States Department of Agriculture.
